# A deep learning approach to investigate the filtration bleb functionality after glaucoma surgery: a preliminary study

**DOI:** 10.1007/s00417-023-06170-6

**Published:** 2023-08-02

**Authors:** Leonardo Mastropasqua, Luca Agnifili, Lorenza Brescia, Michele Figus, Chiara Posarelli, Francesco Oddone, Sara Giammaria, Matteo Sacchi, Marco Pavan, Dante Degli Innocenti, Valentina Olivotto, Stefano L Sensi, Rodolfo Mastropasqua

**Affiliations:** 1 Ophthalmology Clinic, Department of Medicine and Ageing Science, University “G. D’Annunzio” of Chieti-Pescara, Via Dei Vestini Snc, 66100 Chieti, Italy; 2 Ophthalmology Unit, Department of Surgical, Medical, Molecular Pathology and Critical Care Medicine, University of Pisa, Via Roma 67, 56126 Pisa, Italy; 3 IRCCS Fondazione Bietti, Via Livenza, 3, 00198 Rome, Italy; 4 University Eye Clinic, San Giuseppe Hospital, University of Milan, Milan, Italy; 5 Datamantix S.R.L. Artificial Intelligence Company, Via Paolo Sarpi, 14/15, 33100 Udine, Italy; 6 Department of Neuroscience, Imaging and Clinical Sciences (DNISC), “G. d’Annunzio” University of Chieti-Pescara, Via Dei Vestini 31, 66100 Chieti, Italy

**Keywords:** Glaucoma, Filtration surgery, Filtration bleb, Bleb functionality, Surgical outcome, Artificial intelligence, Deep learning

## Abstract

**Purpose:**

To distinguish functioning from failed filtration blebs (FBs) implementing a deep learning (DL) model on slit-lamp images.

**Methods:**

Retrospective, cross-sectional, multicenter study for development and validation of an artificial intelligence classification algorithm. The dataset consisted of 119 post-trabeculectomy FB images of whom we were aware of the surgical outcome. The ground truth labels were annotated and images splitted into three outcome classes: complete (C) or qualified success (Q), and failure (F). Images were prepared implementing various data cleaning and data transformations techniques. A set of DL models were trained using different ResNet architectures as the backbone. Transfer and ensemble learning were then applied to obtain a final combined model. Accuracy, sensitivity, specificity, area under the ROC curve, and area under the precision-recall curve were calculated to evaluate the final model. Kappa coefficient and *P* value on the accuracy measure were used to prove the statistical significance level.

**Results:**

The DL approach reached good results in unraveling FB functionality. Overall, the model accuracy reached a score of 74%, with a sensitivity of 74% and a specificity of 87%. The area under the ROC curve was 0.8, whereas the area under the precision-recall curve was 0.74. The *P* value was equal to 0.00307, and the Kappa coefficient was 0.58.

**Conclusions:**

All considered metrics supported that the final DL model was able to discriminate functioning from failed FBs, with good accuracy. This approach could support clinicians in the patients’ management after glaucoma surgery in absence of adjunctive clinical data.

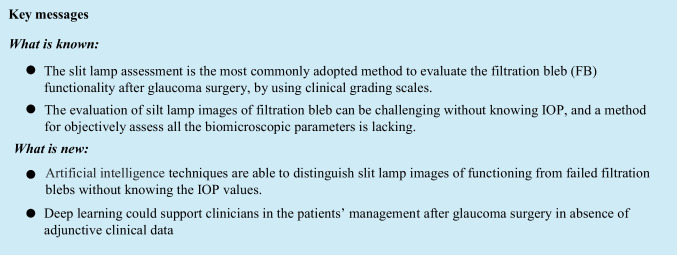

## Introduction

To date, filtration surgery (FS) remains the most performed and effective approach to lower intraocular pressure (IOP) in patients with uncontrolled glaucoma. Among available options, trabeculectomy still represents the gold standard procedure.

FS works by creating an intrascleral fistula between the anterior chamber and subconjunctival space, which leads to the elevation of the conjunctiva at surgical site and the formation of a filtration bleb (FB). The aqueous humor (AH) flows into bleb spaces and then is removed by several routes [1, 2]. Therefore, FB represents the most critical structure after surgery since the long-term success of the procedure depends on its correct development and functionality [3]. Because of this, the accurate evaluation and monitoring of the bleb morphology and function during the postoperative period represent the most crucial steps to reveal in time the earliest signs of surgical failure [4]. Though imaging systems such as anterior segment optical coherence tomography, ultrasound biomicroscopy, or in vivo confocal microscopy can significantly improve the FB evaluation by providing reliable hallmarks of filtration ability along the entire surgical pathway, they require a significant expertise in the execution and interpretation, are time consuming, and not cost effective [5–7]. Therefore, the slit lamp assessment remains the most commonly adopted method to evaluate the FB over time. Different clinical grading scales have been proposed in the attempt to standardize the FB assessment at slit lamp and to improve the definition of surgical outcome [8–12]. These scales consider some morphological FB features such as elevation, height, vascularization of the central and peripheral bleb portions, and the AH leakage, to distinguish functioning from failing or failed cases. Overall, the interobserver agreement of these scales was reported good to excellent [10–12]. Nevertheless, when considering each single bleb feature, some of them present high interobserver agreements, whereas others present low to moderate agreements, or fail to show satisfying levels of agreement [10–12]. In addition, the evaluation of photographs can be challenging for untrained ophthalmologists, with consistency being considerably better between experienced observers compared to inexperienced graders [12]. Therefore, a method to objectively and accurately assess all the biomicroscopic parameters that are useful to define the FB functionality is still lacking. This is of crucial importance to correctly define the FB functionality without knowing the IOP value, as could be the case of a remote image evaluation for telemedicine.

In the last years, artificial intelligence (AI) proved valuable in improving medical imaging interpretation for screenings, precision medicine, and risk assessment in different fields of ophthalmology, including glaucoma [13]. Nevertheless, the application of AI in glaucoma surgery is still very limited, with only one study that used deep learning (DL) (Mask R-CNN) to evaluate FBs after trabeculectomy. This study found that DL is promising in the FB monitoring after surgery but limited its interest in the evaluation of just one parameter, the bleb size, and its correlation with IOP [14].

The aims of this preliminary present study were to exploit AI techniques to distinguish slit lamp images of functioning from failed filtration blebs, by designing and implementing an image classification system. We developed a DL model based on a convolutional neural network (CNN) using a ResNet architecture [15]. To improve the system accuracy, a complex operational pipeline was defined, starting from data transformation techniques to the transfer learning (TL) approach [16, 17]. Finally, to reach higher performances and to obtain more robust results, different models of analysis were combined by using the ensemble learning (EL) [18].

## Methods

### Patients

This was a retrospective, cross-sectional, multicenter Italian study. It was carried out at the University of Chieti-Pescara, University of Pisa, and the Glaucoma Unit of the IRCCS Fondazione Bietti (Rome) for patients’ enrollment, and in collaboration with the Datamantix S.r.l. Artificial Intelligence Company, Udine, Italy, for data analysis. We collected 119 color pictures of 12 o’clock located FBs from Caucasian patients who underwent FS and of whom we were aware of the outcome. FB pictures were provided from the three glaucoma centers of each research unit (50 images from Pisa, 40 from Chieti-Pescara, and 29 from Rome), at the 12th month follow-up.

All patients provided written informed consent prior to enrolment, after explanation of the purpose and possible consequences of the study. The study was prospectively approved by the Institutional Review Board of the Department of Medicine and Ageing Science of the University ‘G. d’Annunzio’ of Chieti-Pescara, Chieti, Italy and adhered to the tenets of the Declaration of Helsinki.

Inclusion criteria for patients were the following: age ≥ 18 years, a diagnosis of open angle glaucoma, an uncontrolled IOP (> 21 mmHg) under maximal tolerated medical therapy and/or progression of the visual field (VF) damage confirmed on three consecutive examinations (Humphrey field analyser (HFA) II 750; Carl Zeiss Meditec Inc., Dublin, CA, USA (30–2 test, full threshold)) before surgery; history of fornix based mitomycin-C (0.02%, 2–3 min) augmented trabeculectomy, performed by one surgeon for each Center (Chieti-Pescara: LM; Pisa: MF; Rome: FO); absence of major intra or postoperative complications. Laser suture lysis, bleb needling, and the postoperative use of antimetabolites were allowed to promote bleb filtration. Trabeculectomy was considered successful when a third reduction of the baseline IOP, with IOP values ≤ 18 mmHg with or without the use of IOP lowering medications, was achieved at 12 months after surgery. Three different outcome classes were defined at the 12th month: (i) complete success (C), which is success without the use of medications; (ii) qualified success (Q), which is success with supplemental medical therapy; and (iii) failure (*F*).

#### Image analysis

**Fig. 1:**
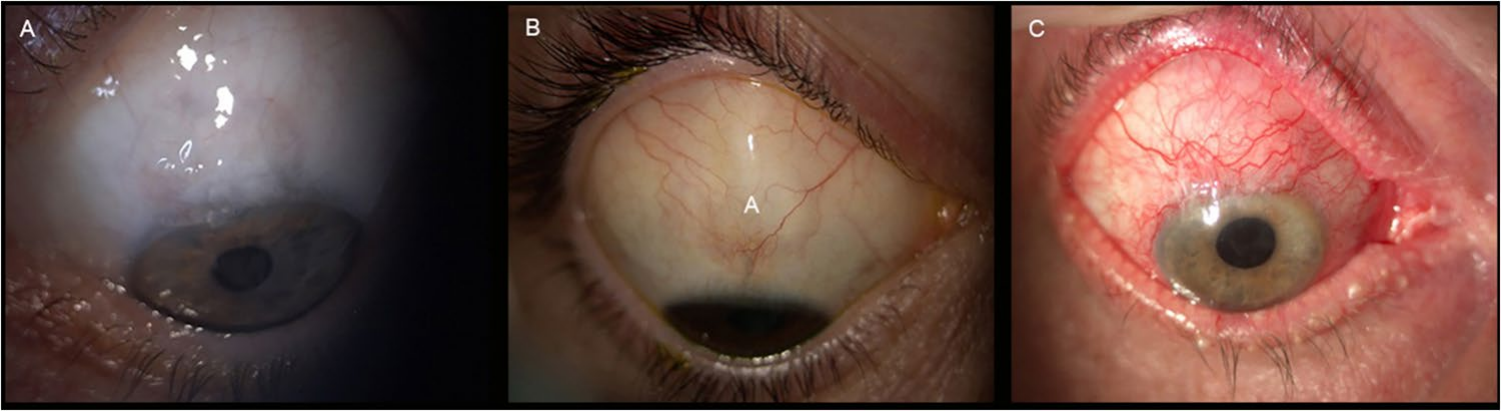
Slit lamp images of the three groups. **A** Complete success (C). FB appears well-elevated and extended on the ocular surface, with indistinct margins and with a multi-cystic feature in the juxta-limbal side; the bleb-wall is hypo-vascularized and transparent. **B** Qualified success (Q). FB appears well-elevated on the ocular surface, shows a circumscribed shape (probably encapsulated in part) with relatively defined margins, and has a normal vascularization; the bleb-wall color indirectly indicates the presence of a thick bleb-wall. **C** Failure (F). FB appears flat, with a dense, and irregularly hyper-vascularized bleb-wall, and with indistinct margins; the entire ocular surface is hyperemic, with eyelids showing signs of Meibomian gland dysfunction

Bleb pictures were taken in different shooting conditions by using the SL 9900 ELITE slit lamp (CSO, Costruzione Strumenti Oftalmici, Firenze, Italy), with the eye in downward gaze to expose at best the surgical site, and gently elevating the upper eyelid. Figure 1 shows representative FB images of the three outcomes classes, extracted from the collected set, taken in different shooting angles and light exposures.

By using an approach based on AI, it is possible to automatically classify a whole image of a FB and get a qualitative response. To proceed with this approach, images require an annotation process which has the purpose of assigning a label to each of them, according to the outcome classes. These labels identify the classification assigned to the surgical outcome, following the three classes C, Q, and F, and are given by experienced glaucoma surgeons. In particular, the images were randomly splitted into several groups, and each of them has been analyzed by glaucoma specialists (LB, CP). LB and CP manually classified each picture considering the image characteristics and other clinical data such as the IOP, the nature and timing of surgery, and the post-surgical therapy (if any). After this first annotation, the images were reviewed by other experienced glaucomatologists (LA, MF, FO) to consider different experts' opinions and avoid misclassification of data. The review of the annotations by various specialists represents a crucial step in the DL process since truth labels are essential both in the learning phase and in the evaluation of the model.

To carry out a qualitative analysis, an image classification task was therefore defined. Problems related to the image classification process are currently solved using DL techniques, a branch of AI which includes the deep neural networks, and in particular, a variant of them designed specifically for image analysis tasks, called CNN. Among the different architectures of these networks, there is a very effective one in the classification of the whole image, which is based on residual block approach and is called ResNet [15]. This network can be implemented in different variants based on the number of layers.

**Fig. 2:**
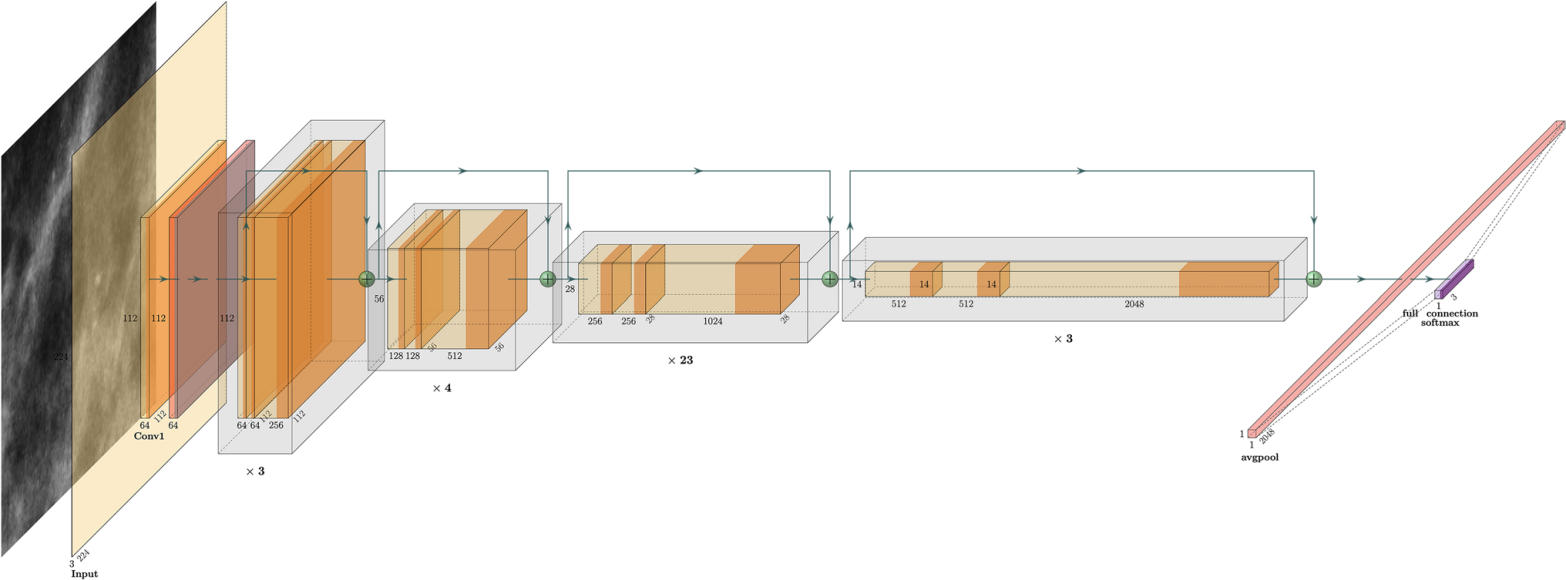
ResNet-101 architecture. Image generated using PlotNeuralNet open-source tool (https://github.com/HarisIqbal88/PlotNeuralNet)

**Fig. 3:**
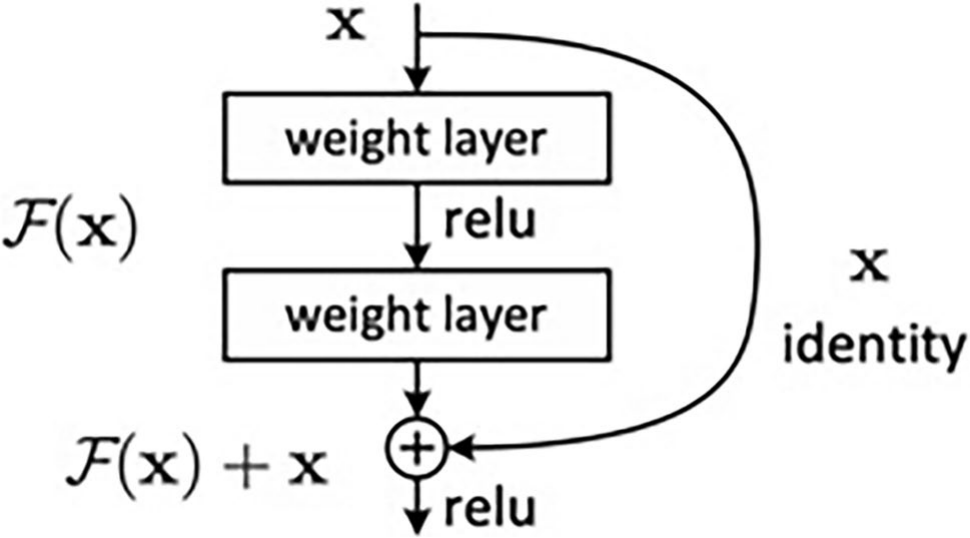
Residual learning: a building block. Image from the original ResNet paper *(*He K, Zhang X, Ren S, Sun J. Deep Residual Learning for Image Recognition. In CVPR. 2016;770–778)

By configuring different numbers of channels and residual blocks in the module, it is possible to create different ResNet models, from the lighter 18 layers, called ResNet-18, to the deeper 152-layer, called ResNet-152. Figure 2 reports the general architecture of ResNet-101 with the aim to show the general structure of the layers that make up the network, regardless of the number of those used. Figure 3 shows the detailed logic of a residual learning block that characterizes this type of network with the shortcut connections.

In this study, three different ResNet architectures were implemented to perform the experiments: ResNet-18, ResNet-34, and ResNet-50. This choice is due to address the tradeoff between network capacity and dataset size. Usually, these models outperform by a significant margin in case the network is deeper and more complex. At the same time, if the dataset is small, a deep architecture can cause overfitting problems and provide poor generalization. For this reason, since the number of images collected was limited, we did not consider ResNet architectures with more than 50 layers. The network capacity of smaller ResNet is sufficient to capture the essential image characteristics to achieve the classification goal.

However, some critical issues made the network training process complicated: (i) the limited number of annotated images to train the system on; (ii) the presence of distracting elements within the images, such as fingers and face masks; (iii) the high variability of conditions in which the studied phenomenon occurred; (iv) the presence of adjacent classes, that may show up with minimal differences between them, which make hard the FB discrimination even by expert glaucomatologists; and (v) the presence of unbalanced outcome classes, with 47% of *C*, 34% of *Q* and 19% of *F*.

**Fig. 4:**
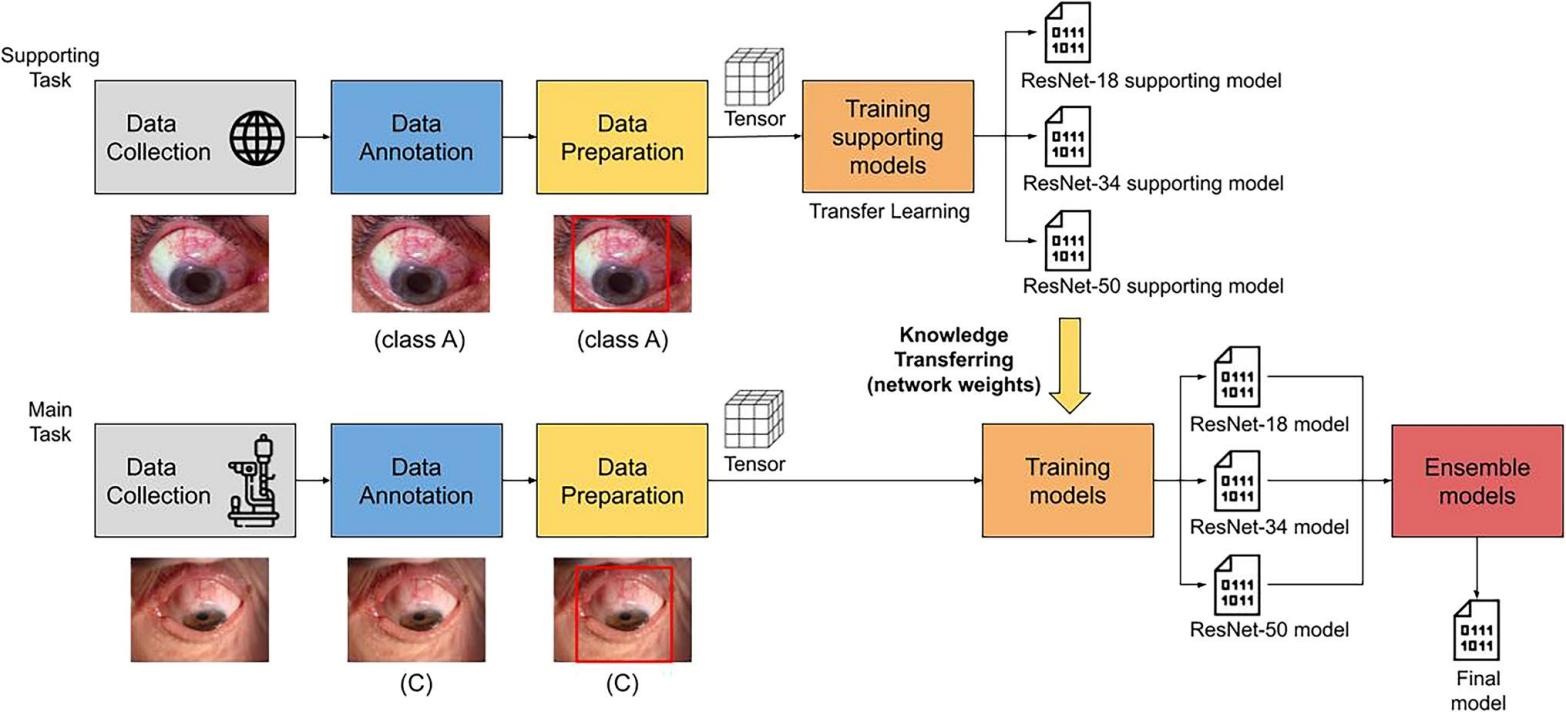
The operational pipeline of the qualitative analysis of functional filtering bleb. The upper part of the figure reports the supporting task defined for the transfer learning technique. The lower part of the figure describes the pipeline for the classification of FB functionality, where the networks are initialized with the knowledge obtained from the supporting task

Therefore, countermeasures were adopted to limit as much as possible these criticisms. In particular, we defined an operational pipeline as follows: (i) data preparation: the collected raw data are transformed into a form that is suitable for further processes and that can improve the model results; (ii) transfer learning: this technique is particularly highly effective when faced with domains with limited data, such as the medical one; and (iii) ensemble learning: by combining multiple models it is possible to reduce the high variance and bias of a single classification model. Figure 4 depicts the whole pipeline from data preparation to the training of a set of models, and to the combination of models in a final one.

All experiments were performed on a workstation specific for AI purposes, based on the NVIDIA v100 GPU with 32 GB memory VRAM configuration. All the phases were developed using Python v3.10 with *PyTorch* v1.12.1 as backend, to train ResNet models exploiting the best modern practices, while different open-source Python libraries, such as *OpenCV*, *scikit-learn*, and *Matplotlib*, were used to implement the data preparation phase, the evaluation of the models and the final analysis of the results.

#### Data preparation

The primary purpose of data preparation is to ensure that the raw data is thoroughly prepared for processing to improve the accuracy and consistency of the analyses. In the following sections, we describe the three steps defined to apply cleaning, transformation, and augmentation processes to data, to address the critical issues.

### Data cleaning

Since images were taken in different shooting conditions, the brightness levels of pictures can be very different. Moreover, since the dataset is very small, few images can strongly affect the model evaluation and the final results. Therefore, a data cleaning process was necessary. Deep analysis has been conducted to identify the images that were too dark or too blurry, and that can neither help the model to improve the classification task, nor be used in the test set to evaluate the prediction. Finally, 9 images were removed from the original collection, ending up with a total of 110 samples to train, validate and test the AI system. On the other hand, we decided to keep in the dataset all the other images that do not exhibit extreme lighting conditions, to enhance the performance of the model. In fact, one of the best practices for building a reliable dataset is to include a sufficient number of images representing the subject of interest in different shooting conditions.

### Data transformation

To improve the performance of an AI model trained on images, transformations to the images contained in the dataset are commonly applied. This step will make the subject of interest more evident, acting on colors, brightness, or even removing parts of the image. Images may contain many distracting elements that can negatively affect the model classification. Basically, these elements can create wrong biases, since the ResNet architecture can start to deeper analyze the distracting elements to classify the surgery outcome, instead of the FB area. Moreover, CNNs usually require identical image sizes to work properly. Due to these reasons, all the images were cropped into squares of size 1024 × 1024 pixels, cutting the distracting elements, and centering the picture on the salient area for the classification. A deep neural network is computationally expensive; therefore, using squared images and keeping image size as low as possible is an adequate strategy. We defined a good tradeoff between image resolution and size to see the graphical information necessary for the prediction task and to guarantee addressable training processes.

### Data augmentation

To increase the amount of data, a set of techniques to generate artificial images can be applied. All these strategies fall within the data augmentation phase. This process helps the improvement of the model accuracy, adding more samples in the training set, and preventing overfitting. Different types of transformations can be applied to the original samples. In this study, geometric transformations (clockwise and anti-clockwise rotations), per-pixel transformations (changes in brightness, contrast, saturation, and color levels), and destructive transformations (increase blur level) were applied to the starting dataset. Therefore, using various combinations of these transformations, we generated a set of different datasets to run several training processes and compare the performance, with the purpose of highlighting the differences caused by the diversity of the images.

The experiments conducted prove that the data preparation phase has a significant impact on final results. In particular, both data cleaning and data transformation processes improved the learning of the model and the accuracy of the predictions. On the other hand, data augmentation techniques did not considerably contribute to solve criticisms, since results obtained after these transformations were equivalent to the baseline models that used original data. On this basis, the first two phases described in this section have been applied to the dataset before proceeding with the training and the evaluation phases, avoiding data augmentation.

#### Transfer learning

Transfer learning helps data scientists to take a blended approach from different models to fine-tune a solution to a specific problem. The sharing of knowledge between two or more different models, transferring knowledge from one to another, can result in a much more accurate and powerful model. In the following steps, we describe a dataset and a task that are different from the main aim of the study, but correlated and relevant in terms of image features. This allows us to get a supporting AI model and exploit the acquired knowledge to run the training of a new one on our main task, starting on a more robust basis.

### Supporting task and dataset

In this study, the images collected are taken using a specific slit lamp. The classification task is difficult since it considers these specific types of images and there are no similar categories in the known public datasets used to pre-train the CNNs. The general idea is to solve a similar task using comparable images, training a network, and then using the obtained knowledge (the neural network weights) to train a different network with the aim of classifying the surgery outcome. Figure 4 depicts this process. By using a web scraping technique, a specific dataset has been created. In particular, the following keywords were selected to perform the search on Google: “glaucoma filtration bleb”, “glaucoma filtering bleb”, “filtering bleb”, “glaucoma”, “conjunctival bleb”, “filtration surgery”, and “trabeculectomy”. Because we were not aware of IOP values and of the use of IOP lowering medications, FB images constituting the supporting dataset were evaluated according to the Indiana Bleb Appearance Grading System (IBAGS) [8]. The IBAGS is a standardized and objective system for grading of FB anatomy and proved valuable and reliable over other grading scales [8, 10]. It contains a set of photographic standards for grading bleb height (H), extent (E), vascularity (V), and leakage with the Seidel test (S). Since images constituting the supporting dataset were not directly taken at slit lamp, the feature S was not considered.

All the images collected were analyzed to remove the not relevant ones and they were labeled by experienced glaucomatologists (LB, CP) according to the IBAGS scales of H, E, and V. The supporting dataset, which includes a total of 147 samples, has been used to train a ResNet model for three classification tasks, with the height (H0–H4), the extent (E0–E4), and the vascularity (V0–V4) intervals as target classes, respectively, to explore which model could be the best candidate to act as supporting model. Since the best results have been achieved for the classification of conjunctival vascularity, the model trained to solve this task has been selected as the base for the next steps.

### Training the supporting model

Since we wanted to exploit the potential of different ResNet architectures, we trained more than one model. In particular, ResNet-18, ResNet-34, and ResNet-50 have been trained using a training set of 118 images, and a validation set of 29 samples. In all the experiments carried out, the image size considered has been 1024 × 1024 pixels, and no other transformations have been applied.

Finally, the weights of these networks have been saved to initialize the training processes for the main task.

#### Ensemble learning

Ensemble learning refers to algorithms that combine the predictions from two or more models to obtain better predictive performance. We adopted this approach because with more than one ResNet model we can leverage the capabilities of the different network variants described in previous sections and elaborate a final classification model by combining results. In such a way, we simulated a sort of request for an opinion to ophthalmologists with different expertise.

### Ensemble settings

To improve the robustness and the capacity of generalization of the AI system, the predictions of three different models have been combined using an ensemble technique. In particular, the soft voting ensemble method has been applied, considering the same weight for each classifier since there are no reasons to set different importance levels. Therefore, the final target label is defined as the class with the greatest sum of weighted confidence levels.

### Training the models

Once the data preparation techniques and the overall workflow have been defined, the final models have been trained. The whole dataset has been split randomly into a training set (60%), a validation set (20%), and a test set (20%). Thanks to the transfer learning approach, the acquired knowledge of the supporting task has been used to initialize a more robust classifier. Moreover, the parameter optimization and the fine-tuning phases have been conducted to achieve a great performance in the FB functionality classification. Therefore, the three networks have been trained using different key parameters. The number of epochs for the ResNet-18, ResNet34, and ResNet-50 have been set to 25, 15, and 30, respectively, while the batch size has been equal to 16 in all cases. As for supporting task models, the three networks have been trained using an Adam optimizer and the CrossEntropyLoss as loss function. We found the more appropriate learning rates for the models and the training phases have been implemented using the technique of discriminative fine-tuning, based on different learning rates for different layers groups [19, 20]. In particular, we considered starting values in the range [1e-6,1e-4]. Moreover, we exploited the stochastic gradient descent with warm restarts (SGDR), to make the training processes more efficient [21]. Finally, the predictions of the three resulting networks were combined using the soft voting ensemble technique.

#### Evaluation and statistical analysis

Student *t* test and chi-square test were used to evaluate pre-operative demographic data and gender differences among groups. To evaluate the AI system, a set of metrics were calculated [22, 23]. Moreover, to provide more details about model performance, we illustrate additional metrics for each target class on the test set.

In particular, we show accuracy, sensitivity, specificity, area under the ROC curve (AUC), and area under the precision-recall curve (AUPR). Since the classes were unbalanced, the overall measures were computed using the micro-average calculation to aggregate all the categories’ contributions.

Some statistical measures, such as the *P* value and the Kappa coefficient, have been examined using the confusion matrix information. In particular, a one-sided binomial test has been computed to evaluate whether the overall accuracy rate is greater than the rate of the largest class, to see if the accuracy is better than the no information rate. Furthermore, the statistical coefficient Kappa allowed us to measure inter-rater agreement for categorical items. It is generally thought to be a more robust measure than simple percent agreement calculation, as it considers the possibility of the agreement occurring by chance. Both *P* value and Kappa are considered helpful for a dataset with unbalanced classes, as in this study [24].

## Results

One hundred and ten images were considered in the analysis: C: 48 cases (44%), Q: 39 (35%), and F: 23 (21%). The demographic and clinical characteristics of patients and the three outcome classes are reported in Table 1. Group F had a significantly higher mean number of postoperative bleb manipulation procedures compared to Groups C and Q during the 1st year after surgery (3.4 ± 0.5, 1.1 ± 0.2, 1.9 ± 0.6, respectively; *p* < 0.05).

**Table 1:** Preoperative demographic data of the three outcome classes

Group	No	Age (years ± sd)	Sex (M/F)	IOP (mmHg ± sd)	MD (db ± sd)	Time on medical therapy (months ± sd)
C	56	68.26 ± 14.8	30/26	29.03 ± 6.51	− 3.85 ± 2.54	84.24 ± 23.31
Q	40	70.3 ± 10.4	22/18	26.7 ± 3.1	− 5.2 ± 1.88	77.31 ± 19.12
F	23	69.69 ± 14.6	10/13	24.53 ± 6.58^†^	− 4.66 ± 3.11	96.12 ± 32.64 *

*SD*, Standard deviation; *IOP*, intraocular pressure; *MD*, mean defect

*p < 0.05 vs. Q; ^†^ < 0.05 vs. C

Different metrics were analyzed to evaluate the model, with results computed on the test set reported below. Considering the total of correct predictions against the whole population of test cases (# true positives/# total images), we reached an overall accuracy score of 74%. Since we are interested to show the performance on three outcome classes, we computed the accuracy for each of them ((# true positives + # true negatives)/# total images). We obtained a score of 87%, 78%, and 83% for classes C, Q, and F, respectively. All these values appear good, considering the specific task of the study and the limits of the collected dataset.

**Fig. 5:**
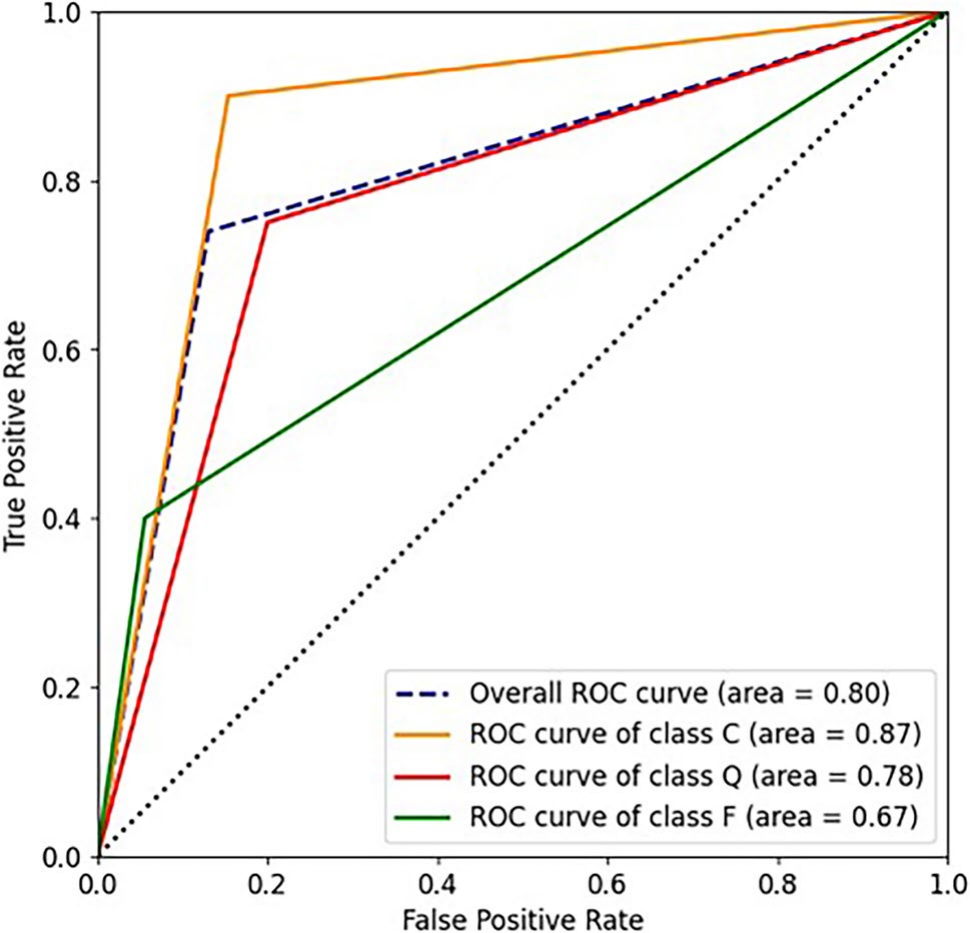
ROC curves

**Fig. 6:**
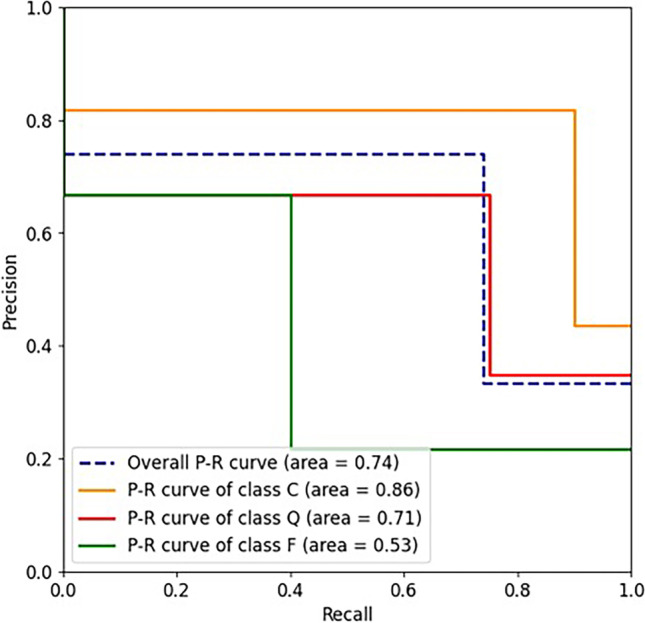
Precision-recall curves

**Fig. 7:**
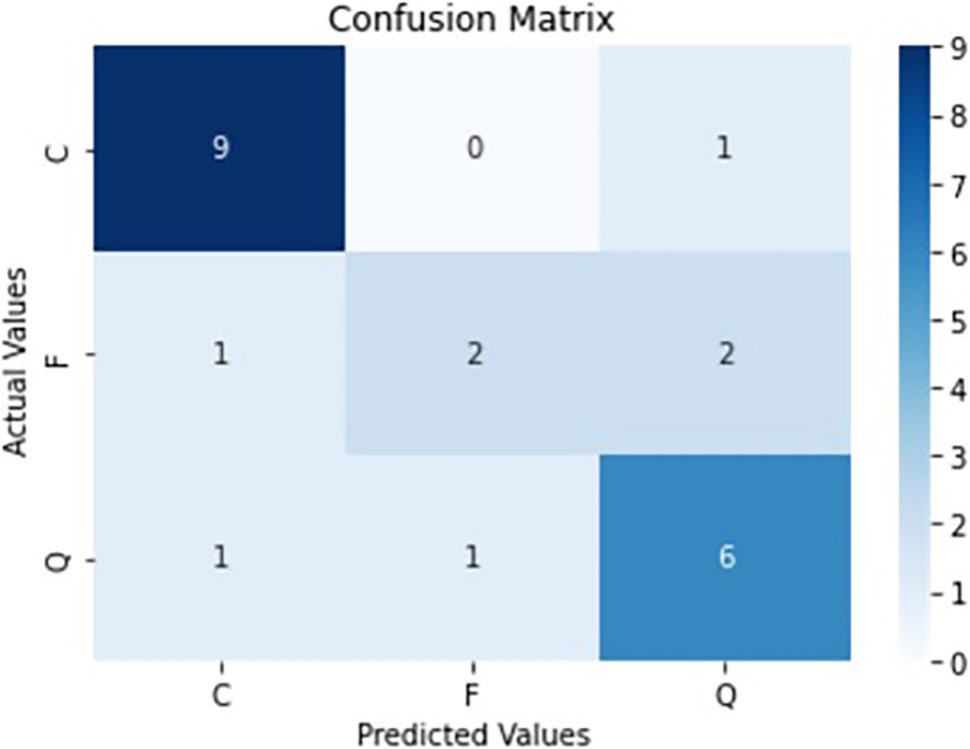
Confusion matrix for the test set data

Moreover, the classification achieved 74% sensitivity and 87% of specificity. As expected, the category with the worst value of sensitivity was the class F, due to the limited number of failures in the original dataset, and the best category was the class C, thanks to the higher number of images in the training set. AUC and AUPR values, 0.8 and 0.74, respectively, highlight how the AI system can discriminate between the three outcome classes (Figs. 5 and 6). Figure 7 shows the confusion matrix, which reports the results of all the predictions against the actual values assigned during the annotation phase.

Table 2, which reports other important metrics per each target class, further documents the satisfactory quality of the system. P-value was equal to 0.00307 (*P* < 0.05) and the Kappa coefficient was 0.58, and these metrics have been computed as described in the Evaluation and Statistical Analysis section. Kappa value is considered “Intermediate to good” for the Fleiss’ benchmark, and “Moderate” for Landis and Koch’s and Altman’s benchmarks [25–27]. Thus, both confirm the statistical significance of the final model.

**Table 2:** Summary of the most important metrics per each target class

Metric	Class C	Class Q	Class F
Accuracy	0.86957	0.78261	0.82609
AUC	0.87308	0.775	0.67222
AUPR	0.85909	0.70833	0.53333
F1-score	0.85714	0.70588	0.5
Adjusted F-score	0.89194	0.78635	0.61409
FPR	0.15385	0.2	0.05556
FNR	0.1	0.25	0.6
TPR (sensitivity)	0.9	0.75	0.4
TNR (specificity)	0.84615	0.8	0.94444
Precision	0.81818	0.66667	0.66667

*AUC* Area under the ROC curve; *AUPR* area under the precision-recall curve; *FPR* false positive rate; *FNR* false negative rate; *TPR* true positive rate; *TNR* true negative rate

**Fig. 8:**
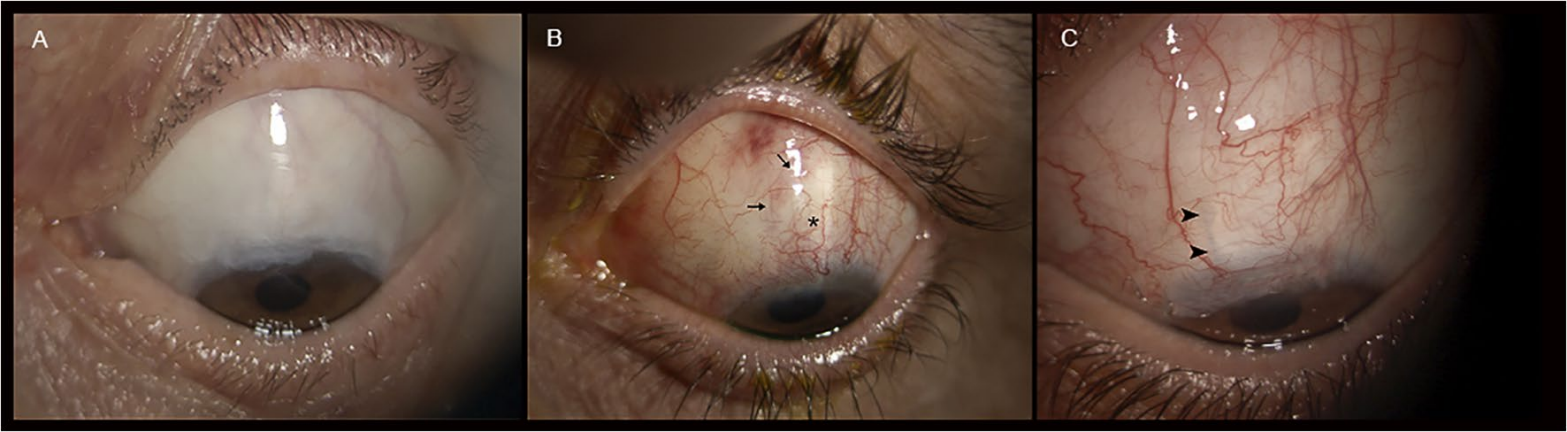
Examples of top loss images. **A** Classified as F, predicted as C. A dense and thick conjunctiva in the iuxta-limbal region may underlie a failed bleb; however, the avascularity and the features of the posterior part of the bleb (conjunctiva slightly elevated and transparent) indicate a contained fibrosis and efficient aqueous humor filtration.** B** Classified as Q, predicted as F. Despite the presence of evident fibrosis and vascularization for the most part of the FB (asterisk), the nasal and posterior peripheral regions of the bleb present signs of filtration with a less vascularized, transparent, and elevated conjunctiva (arrows). **C** Classified as Q, predicted as F. The predominant aspect of the bleb is flat and fibrotic, indicating a failure. However, at a deeper observation, the nasal margin of the scleral flap (arrowhead) is still separated from the surrounding sclera permitting in part the aqueous humor outflow

Moreover, to highlight the main issues of the classification model, we conducted a qualitative analysis of FB images that the AI system misclassified with the highest error (i.e., top losses images). Figure 8 shows some of these images, which appear difficult to classify, given the particular characteristics of FBs. Reasons underlying this arduous classification may depend on the fact that parameters characterizing the FB functionality are in part bidimensional, such as vascularization or surface extension, in part three dimensional, such as the height, and in part dynamic, such as the aqueous humor leakage.

## Discussion

The correct interpretation of the FB function in the routine clinical practice, can be sometimes challenging without knowing IOP, even for glaucoma surgeons with a consolidated expertise.

In the present preliminary study, we developed a DL approach to analyze slit lamp images of FBs according to their known outcome, with the aim to distinguish functioning from failed cases. Even though the number of FB images contained in the dataset was limited and there were three target classes that further challenged the analyses, the final DL model reached good results for all the metrics that were considered.

In more detail, when considering the overall accuracy and the accuracy for each class, the DL model was able to correctly distinguish successes from failures, properly classifying the three outcome classes in two-third of cases (74%). When the accuracy was computed per each single outcome class, the model reached more than four fifths of correct classifications for the C (87%) and F class (83%), and more than three fourths for the Q class (78%). Thus, as evident, excellent results were obtained especially for successful cases, either for the class C or Q. On the other hand, since the outcome class F was less represented, measures of the model were less performant, with AUC, AUPR, and F1-score values lower than 20%, 32%, and 36%, respectively, compared to outcome class C. Nevertheless, the accuracy and the specificity of the class of failures were excellent (83% and 94%, respectively), indicating that the model learnt the major critical features that characterize failures.

When looking at sensitivity, the system achieved good performance in classifying classes C and Q, whereas it was slightly confused for the class F, as highlighted by metrics shown in Table 2. Regarding specificity, high values confirmed high true negative rates for each class. All these results were achieved thanks to the design of a complex operational pipeline that combined several advanced techniques, which permitted advancements compared to simpler applications of AI with CNN in glaucoma or other ocular diseases [28, 29].

Therefore, in a general critical interpretation, we may state that this pioneering study is particularly promising in the FB assessment since one may expect that measures could further improve by adding more samples to the dataset.

To date, just a previous study used DL to investigate FBs after glaucoma surgery, but in a less articulated way and focusing the attention just on the bleb size [14]. In this study, Wang et al. analyzed successful FBs without discriminating between complete or qualified success, and without considering failures. Thus, the authors were unable to provide information on the ability of the DL model in discriminating complete from qualified successes, and successes from failures, which is the main clinical need in the post-surgical follow-up of patients.

In comparison with the extremely poor literature that explored this topic, our approach presents some important strengths.

First, we addressed a more complex task by designing and implementing an AI system which was able to make qualitative analysis on the whole FB image but was also able to provide some indirect geometric information derived from quantitative parameters (size, height, surface). Though AI cannot directly yield quantitative data since it looks at the whole image, the evaluation of pixels characteristics (such as color and position) constituting the FB image may provide additional quantitative information. Thus, three-dimensional features essential to evaluate the FB functionality in a more comprehensive way, such the bleb elevation, can be indirectly derived thanks to the graphical information embedded in two-dimensional images. The good model performance led us to hypothesize that a significant set of features has been extracted during the training process. Due to the black-box nature of the AI systems, we have no information about this aspect which, therefore, should be evaluated in further studies.

Moreover, our approach allowed us to carry out analysis of the FB without the aid of external data or further processing of parts of the image. This represents a significant aspect since it could open considerations on the use of the AI in the field of telemedicine after glaucoma surgery which, to date, has never been explored. As the COVID-19 pandemic brought to our attention, the possibility to remotely monitor patients with teleconsultations represents an important and unmet issue in chronic diseases requiring frequent controls, such as glaucoma [30, 31]. Whether our results will be confirmed in further larger studies, DL models could help clinicians in assessing FB functionality by analyzing homemade pictures of FBs, in patients who cannot attend medical consultations with regularity.

Second, all the applied techniques, such as data preparation, transfer, and ensemble learning, improved the accuracy of the classifier and the overall robustness of the system. In fact, without the implementation of all the phases described in the pipeline, the evaluation of the classification and the statistical measures would have been worse. Finally, our model was not only a binary classification between successes and failures, but allowed us to distinguish among three target classes, thus permitting a more accurate prediction of the surgical outcomes. In support of this, measures showed that the final DL model performs well for each outcome class, and it is also able to distinguish adjacent categories which, on the other hand, represent the main reasons leading the model to misclassify.

This is a crucial point since our AI system misclassified some FB images with a higher error than the other classifications. When reviewing in detail these images, we noticed that they were particularly hard to classify without adjunctive clinical information such as IOP, also for expert glaucomatologists. Generally, this occurs when FBs present a mixed morphology in which coexist favorable and unfavorable macroscopic features (Fig. 8). Some clinical features of the bleb-wall such as color, transparency, thickness (indirectly derived from the tissue transparency), and margins profile, may be in certain cases not particularly defined. In these circumstances, the FB may concomitantly show characteristics of good functionality along with features indicating failure. Thus, since the predominant feature could not be easily identifiable, clinicians and, therefore, also the AI system, can be confused and make mistakes in interpreting the bleb filtration ability.

On the other hand, we may hypothesize that other morphological bleb features such as vascularization, extension or elevation could be better exploited by the AI system to correctly classify the FB functionality.

All these considerations highlight the complexity of the investigated task and should be intended as a strong point in favor of our model, since it wrongly classifies ambiguous images that need a deeper analysis and external patients’ data. Nevertheless, since the black box nature of AI present significant limitations, the explainability of the present AI system should be investigated in future works.

The present study, since is a preliminary investigation on a new topic, presents some significant limitations. As detailed in the methods section, there were some critical issues that complicated the AI learning process. However, the particular approach (Fig. 4) and the applied techniques we adopted, improved the final performance of the model compared to baseline experiments permitting to solve in large part criticisms.

As already stated, the number of images in the dataset was limited, this generating critical issues both in the training and in evaluation steps. In particular, the lack of an optimal number of images hindered learning all the useful details needed for the classification, leading to a poor generalization. Moreover, the target categories were not equally represented in the dataset, causing the typical issues related to the unbalanced classes. In this case, it is pivotal to verify that the model does not create any type of bias in favor of the better represented classes. Thanks to the data preparation phase and the presence of three research centers in collecting the dataset, the higher degree of diversification in the case selection helped to mitigate this issue. Furthermore, the statistical metrics proved that the accuracy of the model was significant.

With the aim to make the dataset more robust and increase the overall accuracy of the AI system, upcoming works have been planned to obtain additional images, paying attention to improving the class balancing. In addition, to have a more precise focus on the region of interest, investigation on more sophisticated image cropping techniques will be conducted. Finally, combinations of FB imaging with demographic and clinical data will be explored, to evaluate possible correlations and new algorithms.

In conclusion, this study firstly investigated a potential application of the AI in the FB functionality assessment after glaucoma surgery. Our results, despite preliminary and requiring further confirmation, suggest that AI could be a new promising tool, potentially capable of improving the FB evaluation regardless of clinical data. Because telemedicine is rapidly growing, these preliminary findings may contribute to better defining the potential area of application of the teleglaucoma after surgery, especially in the regular patient follow-up, when hospital or outpatient consultations are not feasible.
